# General cognitive but not mathematic abilities predict very preterm and healthy term born adults’ wealth

**DOI:** 10.1371/journal.pone.0212789

**Published:** 2019-03-13

**Authors:** Julia Jaekel, Nicole Baumann, Peter Bartmann, Dieter Wolke

**Affiliations:** 1 Department of Child and Family Studies, University of Tennessee, Knoxville, Tennessee, United States of America; 2 Department of Psychology, University of Warwick, Coventry, United Kingdom; 3 Neonatology, University Hospital Bonn, Bonn, Germany; 4 Division of Mental Health and Wellbeing, Warwick Medical School, University of Warwick, Coventry, United Kingdom; University of Pécs Medical School, HUNGARY

## Abstract

**Objective:**

Very preterm (<32 weeks gestation; VP) and/or very low birth weight (<1500g; VLBW) children often have cognitive and mathematic difficulties. It is unknown whether VP/VLBW children’s frequent mathematic problems significantly add to the burden of negative life-course consequences over and above effects of more general cognitive deficits. Our aim was to determine whether negative consequences of VP/VLBW versus healthy term birth on adult wealth are mediated by mathematic abilities in childhood, or rather explained by more general cognitive abilities.

**Methods:**

193 VP/VLBW and 217 healthy term comparison participants were studied prospectively from birth to adulthood as part of a geographically defined study in Bavaria (South Germany). Mathematic and general cognitive abilities were assessed at 8 years with standardized tests; wealth information was assessed at 26 years with a structured interview and summarized into a comprehensive index score. All scores were *z*-standardized.

**Results:**

At 8 years, VP/VLBW (*n* = 193, 52.3% male) had lower mathematic and general cognitive abilities than healthy term comparison children (*n* = 217, 47.0% male). At 26 years, VP/VLBW had accumulated significantly lower overall wealth than term born comparison adults (-0.57 (1.08) versus -0.01 (1.00), mean difference 0.56 [0.36–0.77], *p* < .001). Structural equation modeling confirmed that VP/VLBW birth (*β* = -.13, *p* = .022) and childhood IQ (*β* = .24, *p* < .001) both directly predicted adult wealth, but math did not (*β* = .05, *p* = .413). Analyses were controlled for small-for-gestational-age (SGA) birth, child sex, and family socioeconomic status.

**Conclusion:**

This longitudinal study from birth to adulthood shows that VP/VLBW survivors’ general cognitive rather than specific mathematic problems explain their diminished life-course success. These findings are important in order to design effective interventions at school age that reduce the burden of prematurity for those individuals who were born at highest neonatal risk.

## Introduction

Very preterm (<32 weeks gestational age, VP) and very low birth weight (<1500 g, VLBW) children have a highly increased risk for academic underachievement, in particular in mathematics [1–4], which has partly been attributed to underlying deficits in attention and executive function [5–8]. It remains controversial whether these mathematic deficits are domain specific [9] or rather explained by global cognitive problems [10].

Population studies have shown that early arithmetic abilities predict long-term academic achievement [11–13] and poor mathematical skills are associated with lower wages, more frequent periods of unemployment, reduced employment opportunities, and lower rates of promotion [14–16]. A number of publications from large datasets such as the British Cohort Study (BCS70) and the National Child Development Study (NCDS) have documented the life-course consequences of basic mathematic skills [17–19] and how these are influenced by cognitive development [20] and environmental risk over time [21]. All these studies have consistently shown that low mathematic skills have uniquely detrimental effects on adult economic success [19].

In addition to life-course studies of normal population samples, recent research on VP/VLBW individuals followed from childhood into adulthood has found that neurodevelopmental and academic problems continue after adolescence, and decreased economic success has also been noted [22–26]. Scandinavian registry-based studies have also shown decreased wealth [27–29] and lower job-related success among preterm born adults [27]. One study has documented strong effects of moderately and late preterm (32–36 weeks gestation, MLP) children’s math achievement on wealth at 42 years, i.e. mathematic attainment uniquely predicted adult wealth after accounting for general intelligence, reading attainment, family socioeconomic status (SES), and various other confounders [30]. This suggests that the pathway from MLP birth to low adult economic success may be specifically mediated by low mathematic attainment in childhood. Compared with MLP, VP/VLBW individuals however are born at more severe neurodevelopmental risk and more often suffer from medical complications and subsequent neurocognitive problems [31–34]. Thus, apart from specific math deficits, VP/VLBW often have multiple cognitive impairments [31, 34]. Research has yet to determine whether VP/VLBW children’s frequent mathematic problems significantly add to their burden of negative life-course consequences over and above the effects of more general cognitive deficits. The aim of this study was to investigate whether negative consequences of VP/VLBW versus healthy term birth on adult wealth are mediated by mathematic abilities in childhood, or rather explained by more general cognitive abilities.

## Materials and methods

Data were collected as part of the prospective Bavarian Longitudinal Study (BLS) [35], a geographically defined whole-population sample of VP/VLBW and term control individuals in Germany. Participating parents were approached within 48 hours of the infant’s hospital admission and were included in the study once they had given written consent for their child to participate. Initial ethical approval was obtained from the University of Munich Children’s Hospital Ethics committee and again in 2009 from the Ethical Board of the University Hospital Bonn, Germany (reference #159/09). All adult participants gave fully informed written consent.

### Participants

Of 70,600 children born in South Bavaria during a 15-month period in 1985/86, 682 were VP (<32 weeks gestation) or VLBW infants (<1,500g), or both. Of these, 411 were eligible for longitudinal follow-up, 14 had severe disability and were unable to be interviewed, and 193/397 (49%) participated at both 8 and 26 years and had complete datasets. The BLS VP/VLBW participants did not differ from adults who dropped out in terms of gestational age, birth weight, duration of hospitalization, sex, maternal age, parental marital status, and childhood cognitive scores, but they had fewer prenatal complications and were more often of higher SES [34].

Healthy term infants who were born in the same obstetric hospitals during the same period were recruited as comparisons. Of an initial sample of 916 children alive at 6 years, 350 were randomly selected within the stratification variables sex and family SES to be comparable to the VP/VLBW sample. Of these, 308 were eligible for longitudinal follow-up assessments, and 217 (70%) participated at both 8 and 26 years. The comparison participants assessed at the 26 year follow-up did not differ from those lost to follow-up in terms of neonatal characteristics but they more often had higher SES, older mothers and higher childhood cognitive scores [34].

### Procedure

Details of peri- and neonatal data have been described elsewhere [35] and are only briefly outlined here. At both 8 and 26 years, participants were assessed for one whole day including cognitive testing and detailed interviews. All assessors and raters were blind to group membership.

### Measures

#### Neonatal variables

Gestational age (GA) was determined from maternal reports of the last menstrual period and serial ultrasounds during pregnancy. Birth weight was documented in the birth records. Infants were classified as small for gestational age (SGA) if they weighed less than the sex specific 10^th^ percentile for their respective GA according to the national standard weight charts (1985–1986) [36].

#### Socioeconomic status (SES)

Information was obtained by standardized interviews within the first 10 days of life. Family SES was computed as a weighted composite score derived from the occupation of the self-identified head of each family together with the highest educational qualification held by either parent [37]. The sum of these three scores was divided by three and recoded into six predefined categories (1 = lowest to 6 = highest).

#### Assessment of mathematic and general cognitive abilities

At age 8 years, children were administered a comprehensive mathematic test [10, 38]. Test tasks were presented in book form with 79 items assessing numerical estimations, calculation, reasoning, and mental rotation abilities. Item responses were scored for accuracy and subscale scores were then summed into a comprehensive total score representing general mathematic abilities. Cognitive abilities were assessed with the German version of the Kaufman Assessment Battery for Children, K-ABC [39]. In the K-ABC, intelligence is measured as a composite score (Mental Processing Component; MPC) based on eight subtests from the Sequential (3 subtests) and Simultaneous (5 subtests) Processing Scales, tapping general cognitive functioning (i.e., Number recall, Hand movements, Word order, Gestalt closure, Matrix analogies, Triangles, Spatial memory, and Photo series, respectively). Reliability is good (.83-.98, split-half method) and construct validity is high (e.g. correlation of .70 with the WISC-R total score).

#### Wealth at 26 years

Information on occupational success and wealth was obtained using a range of interviews and questionnaires. Critical items were selected from these assessments including no own income, unemployment, social benefits, part time work, frequent job changes, not living independently, low educational qualifications, failure to honor financial obligations, impoverishment, as well as restrictions in occupation and economic self-sufficiency. Items were summarized into a comprehensive wealth index score (S1 Table) that was reverse coded for further analysis (i.e., higher scores represent more wealth) analogue to previous summary scores [40, 41].

### Statistical analyses

Data was analyzed with SPSS 24 and Amos 24. We used structural equation modeling (SEM) to answer our research question, whether negative consequences of VP/VLBW versus healthy term birth on adult wealth were mediated by mathematic abilities in childhood or rather explained by more general cognitive abilities. All analyses were adjusted for child sex, SGA birth, and family socio-economic status at birth.

## Results

Table 1 shows that, by definition, VP/VLBW participants were born at lower gestational age and birth weight and more often small for gestational age (SGA) than healthy term born comparisons. There were no group differences with regard to sex, but VP/VLBW adult participants had been born into families of lower SES than term comparisons. At age 8 years, VP/VLBW children had lower mathematic and general cognitive abilities (see Table 1 for details). At age 26 years, VP/VLBW had accumulated significantly lower overall wealth than term born comparison adults.

**Table 1:** Descriptive characteristics of the VP/VLBW and term participants from birth to age 26 years.

	VP/VLBW (*n* = 193)	Term comparisons (*n* = 217)	*Mean difference* *(95% CI)*	*p*
Birth weight (g)	1343 (323)	3363 (447)	2020 (1945–2095)	< .001
Gestational age (weeks)	30.66 (2.24)	39.59 (1.11)	8.92 (8.57–9.27)	< .001
SGA birth (%)	34.5	9.1	5.24^a^	< .001
Sex (% male)	52.3	47.0	1.16^a^	.282
Family SES at birth	3.52 (1.48)	3.83 (1.51)	0.31 (0.01–0.61)	.044
Mathematic abilities at 8 years ^b^	-.72 (1.16)	.14 (.97)	.86 (.65–1.07)	< .001
General cognitive abilities at 8 years ^b^	-.73 (1.34)	.11 (.99)	.85 (.62–1.08)	< .001
Wealth Score at 26 years ^b^^,^ ^c^	-.57 (1.08)	-.01 (1.00)	.56 (.36 - .77)	< .001

Please note: Data are presented as mean (standard deviation) if not indicated otherwise.

^a^*χ*^*2*^*-*value;

^b^ *z*-standardized according to term comparisons, age at assessment was the same in both groups;

^c^ reverse coded

**Fig 1:**
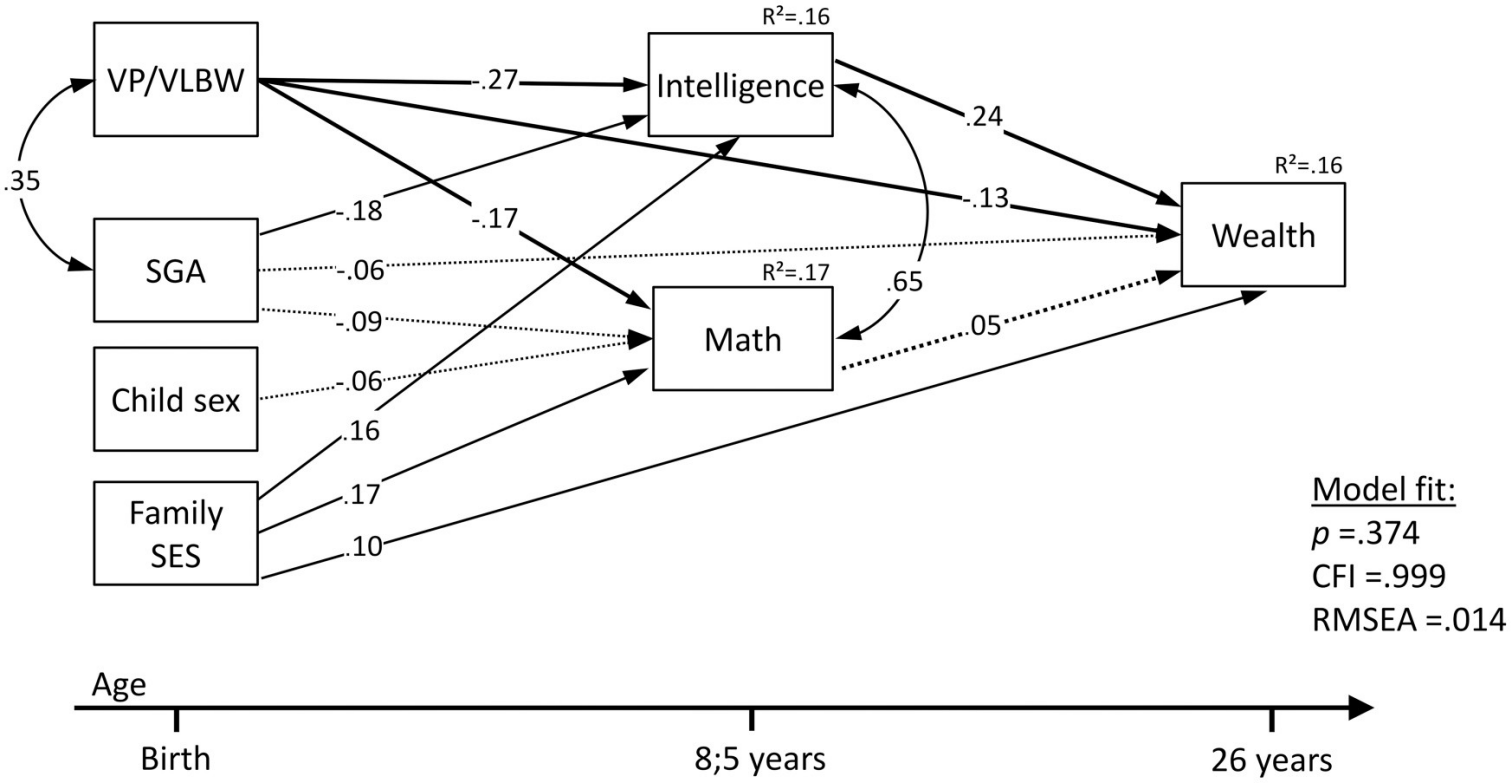
Structural equation model showing effects of VP/VLBW birth, intelligence, and math on adult wealth (*N* = 410).

**Fig 2:**
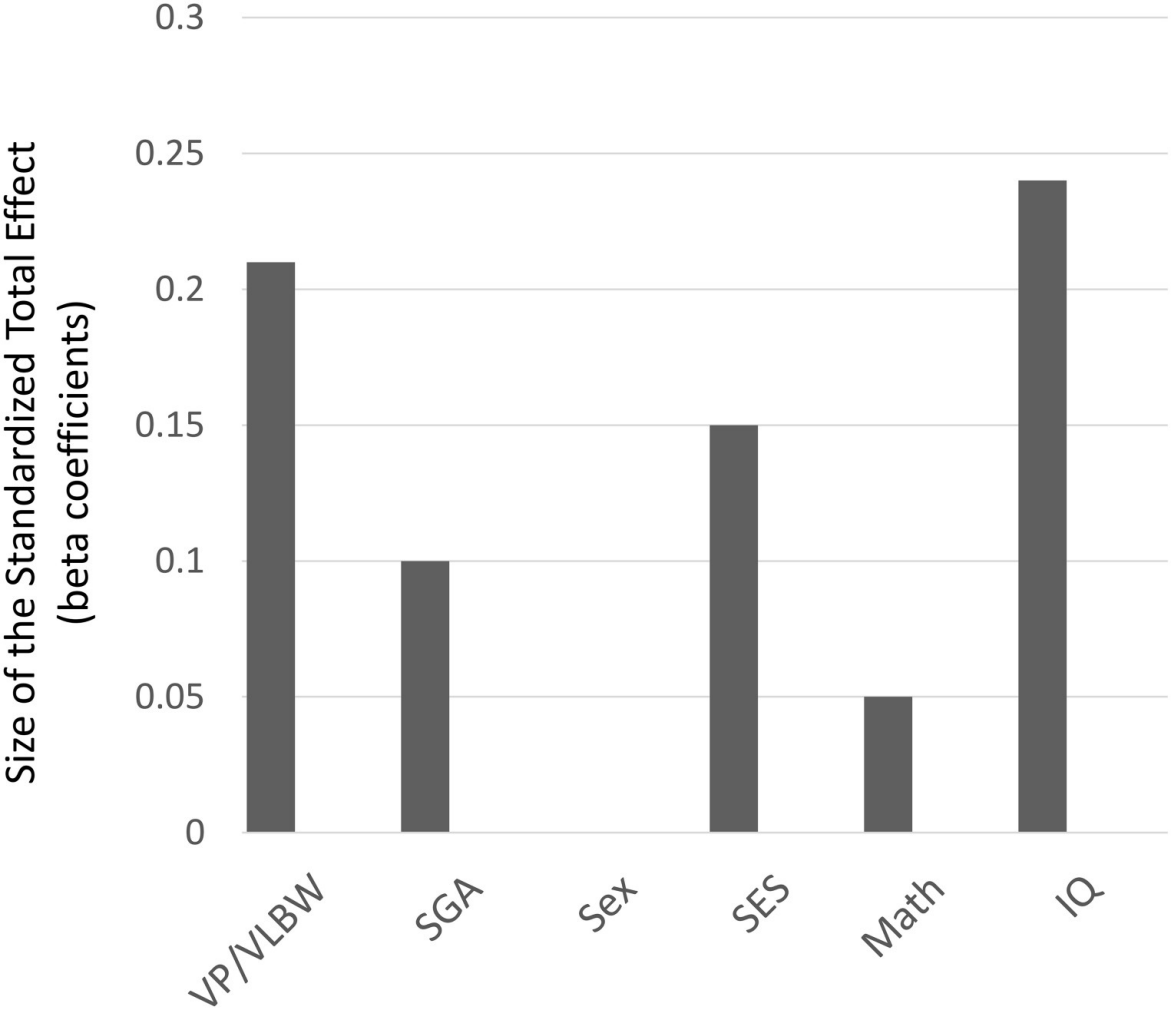
Standardized total (direct + indirect) effects of predictors in the model on VP/VLBW and term adults’ wealth at 26 years (*N* = 410). Please note: Effects are corrected for each other and confounders; for comparison purposes, the strength of each total effect size is shown, irrespective of its positive or negative direction in the model.

Structural equation modeling (Fig 1) showed that VP/VLBW birth and childhood IQ both predicted adult wealth, but math did not. Overall, the model explained 16% of the variation in adult wealth. Model fit was excellent with *χ*^*2*^ = 7.55, *p* = .374, CFI = .999, and RMSEA = .014. Fig 2 shows the total (combined direct and indirect) effects for all the predictors in the model on adult wealth. These effects are unique (i.e., corrected for each other and confounders). With standardized total effect sizes above 0.2, IQ at 8 years (0.24) and VP/VLBW birth (-0.21) were most predictive of wealth at 26 years. The indirect effect of VP/VLBW birth on adult wealth (-0.08; via childhood math and IQ) was not significant, thus mediation was not confirmed.

## Discussion

This prospective 26 year longitudinal study shows that VP/VLBW birth and childhood IQ both independently and directly predict adult wealth, but childhood math does not seem to add to the predictive effects of these two major influences. Previous findings have documented the specific importance of childhood math for adult wealth in moderately and late preterm individuals [30]. In contrast, our results suggest that general cognitive rather than mathematic problems may put VP/VLBW children on a trajectory of economic underachievement and diminished life-course success. Thus, specific math problems may have less of an impact on later wealth considering VP/VLBW individuals’ multiple neurocognitive difficulties. This finding adds to emerging evidence of life-long wide-spread changes in VP/VLBW’s brain networks that are associated with cognitive deficits in adulthood [42–45], and supports previous findings of multiple cognitive rather than specific problems after VP/VLBW birth [34].

Recently, studies have started exploring the neural mechanisms underlying math performance in young adults born preterm and reported altered fronto-parietal activity [43, 46] whereas frontal brain activation is most relevant for general cognitive function [47, 48]. There is additional new evidence that cholinergic basal forebrain integrity may be reduced in those born VP/VLBW and related to low general IQ [49]. This recent finding may open the opportunity for intervention, either by choline supplementation perinatally, dietary or pharmacologically with procholinergic drugs to improve brain development and cognitive development in VP/VLBW children [50], but this is still in the far away future.

Although recent studies have shown that delivery at any gestation other than full term may represent a risk for adverse neurocognitive outcomes [2, 51], the trajectories from neonatal risk through childhood cognitive and academic functioning to life-course success may be different depending on the timing and severity of gestational insults. While VP/VLBW children are at high risk for adverse outcomes, this relationship is not linear across the whole range of gestation: We have previously shown that preterm birth has significant adverse effects on basic mathematic processing following birth at all gestations <36 weeks and on IQ and mathematic attainment <34 weeks of gestational age [4], while adverse effects are generally stronger with lower gestation at birth (i.e., non-linear effect). Moreover, while studies consistently show that VP/VLBW individuals are at high risk for learning disabilities, these are usually comorbid with intellectual impairments [52]. Altogether, the verdict is still open whether VP/VLBW birth puts children at specifically increased risk for mathematic impairments and dyscalculia [1, 3, 53] or whether this risk is explained by their global cognitive difficulties [6, 10]. Our new results add to our understanding of the ‘very preterm phenotype’, i.e. long-term outcomes after preterm birth, suggesting that specific math problems may have less of an impact on later economic success than other factors among those born at highest risk, while there may be different profiles among those born MLP [54]. Future studies may consider VP/VLBW individuals’ multiple problems that extend beyond general cognition [34], such as attention [55], social [56], and emotional difficulties [57–59], in order to investigate the interplay of mechanisms that determine life-course success. We would like to specifically point out that the aim of these current analyses was to assess the competing effects of math versus general IQ in predicting adult economic success, thus we did not include potential additional developmental influences such as attention regulation and executive functions that may further contribute to life-course outcomes after VP/VLBW birth.

No matter whether their functional deficits are global or specific, many VP/VLBW individuals require continuous educational support across various areas of development in order to succeed in school and in life. Most VP/VLBW children have multiple and complex learning difficulties [52] and attend mainstream schools, thus teachers are responsible for supporting them in class. However, teachers may lack formal training about how to appropriately facilitate preterm children’s learning progress [60] and it may be challenging to alleviate the consequences of general cognitive impairments in children’s daily lives. In the last decade, tentative evidence started emerging that an adaptive computerized training program may improve the working memory capacity of extremely LBW preschool children [61] and adolescents [62]. However, transfer effects of cognitive training on long-term academic achievement were not evaluated until two years ago, when new findings from a large population-based Australian RCT showed that academic outcomes of children with low working memory were not improved by working memory training [63]. To date, it is not clear to what extent VP/VLBW children’s long-term developmental trajectories may be influenced with training [64, 65], but life-course studies such as the current one can provide important pointers for the development of effective interventions. Interdisciplinary collaboration and multi-method frameworks will be needed to address different functions that underlie preterm individuals’ complex problems.

This is the first longitudinal study testing independent effects of childhood IQ and math on adults’ wealth using a large, prospectively defined, whole-population sample of VP/VLBW and term comparison individuals born in the same obstetric hospitals at the same time. In total, 58% of the eligible VP/VLBW and term comparisons recruited at birth had complete data at both age 8 and 26 years, however, the dropout was not random as low SES families were less likely to continue participation. As a result, our findings may not generalize to cohorts of preterm children from high social risk backgrounds. However, social factors are a major reason for dropout in most longitudinal studies [66, 67], and our analyses were controlled for family SES at birth. Structural equation model fit values indicated that the pathways shown here very accurately reflect the true developmental mechanisms in our population, and 16% of the total variation in adult wealth was explained, a substantial amount considering the time passed between assessments as well as the ecological validity and comprehensiveness of this outcome variable. Different from previous evidence suggesting the specific importance of childhood math skills for adult success [18, 30], our results show that IQ is a highly significant predictor of adult wealth, as is VP/VLBW birth. In our model, we combined healthy term and VP/VLBW individuals into one sample, nevertheless SEM fit values indicate its validity for both groups. Thus, although our findings may differ from previous studies, they offer new empirical evidence to inform scientific discourse.

In conclusion, our results show that VP/VLBW survivors’ general cognitive problems rather than specific mathematic deficits explain their diminished life-course success. These findings can help inform the design of follow-up and intervention services to reduce the burden of prematurity for those individuals that were born at highest neonatal risk.

## Supporting information

S1 Table (DOCX)
