# Supplementary material for: General cognitive but not mathematic abilities predict very preterm and healthy term born adults’ wealth
Source: PLoS One. 2019 Mar 13;14(3):e0212789. doi: 10.1371/journal.pone.0212789 (PMC6415831; doi:10.1371/journal.pone.0212789)
Supplement: S1 Table — (DOCX) [file pone.0212789.s001.docx]

| **S1 Table.** **Description of indicators for adulthood Wealth Index Score at 26 years of age.** | | |
| --- | --- | --- |
|  | **VP/VLBW (*n* = 193)** | **Term comparisons (*n* = 217)** |
| Receives social benefits ^a^ | 3.2% | 0.5% |
| Receives allowance from parents or others ^a^ | 18.5% | 21.3% |
| Being unemployed at present or in the past or being without occupation ^a^ | 43.5% | 30.0% |
| Working less than 17 hours per week (excluding students) ^a^ | 0.5% | 0.9% |
| Having had more than five jobs ^a^ | 3.2% | 9.3% |
| Lives at parents’/grandparents’ house, in a home, or in a sheltered accommodation ^a^ | 38.3% | 24.0% |
| No secondary school or profession oriented educational qualification ^a^ | 58.0% | 29.0% |
| Sometimes or often failing to pay debts or meet other financial responsibilities ^b^ | 5.1% | 2.8% |
| Relative poverty (lower threshold net income: € 981) [1]^a^ | 31.0% | 28.2% |
| Health limits work or leisure activities OR has just about enough money to make ends meet ^c^ | 33.0% | 25.9% |
| ^a^ Derived from Life Course Interview; please note that items refer to the current situation or the month before the assessment  ^b^ derived from Young Adult Self Report (YASR) [2]  ^c^ derived from the London Handicap Scale [3] | | |

S1 Table References

1. Grabka MM, Goebel J, Schupp J. Höhepunkt der Einkommensungleichheit in Deutschland überschritten? [Has income inequality spiked in Germany?]. Berlin: DIW Berlin — Deutsches Institut für Wirtschaftsforschung e. V.; 2012.

2. Achenbach TM. Manual for the Young Adult Selt-Report and Young Adult Behavior Checklist. Burlington, VT: University of Vermont, Department of Psychiatry; 1997.

3. Harwood RH, Rogers A, Dickinson E, Ebrahim S. Measuring handicap: the London Handicap Scale, a new outcome measure for chronic disease. Quality in Health Care. 1994;3(1):11-6.
